# 1282. Ceftolozane/Tazobactam Use in Critically Ill Patients Receiving Intermittent or Continuous Renal Replacement Therapy

**DOI:** 10.1093/ofid/ofab466.1474

**Published:** 2021-12-04

**Authors:** Molly Henry, Laura A Puzniak, Kelly Harris, Trevor C Van Schooneveld, Trevor C Van Schooneveld, Scott J Bergman, Scott J Bergman

**Affiliations:** 1 Nebraska Medicine, Treynor, Iowa; 2 Merck & Co., Inc., Kenilworth, New Jersey; 3 Merck & Co. Inc, Kenilworth, New Jersey; 4 University of Nebraska Medical Center, Omaha, NE

## Abstract

**Background:**

Our hospital recommends ceftolozane/tazobactam (CT) as a broad-spectrum agent for treatment of Gram-negative bacilli in patients with a recent or current multidrug resistant (MDR) Pseudomonas infection. CT is utilized in patients who are on renal replacement therapy (RRT) yet little data exist on the efficacy in this population. Currently there are no FDA-approved dosing recommendations for patients on continuous veno-venous hemodialysis (CVVHD). The purpose of this study was to describe the indications, dosing and outcomes of patients on CT while receiving RRT.

**Methods:**

All patients receiving CT from 2015-20 were included if on RRT, either CVVHD or intermittent hemodialysis (iHD). Clinical success was defined as the absence of pre-treatment signs/symptoms and/or no escalated antibiotic treatment within 48 hours of completing therapy. 30-day mortality was defined as death from any cause within 30 days of CT completion. Patients treated after 2019 approval of higher dosing for hospital-associated/ventilator-acquired pneumonia (HAP/VAP) were noted.

**Results:**

17 patients received 24 courses of CT while on RRT, 9 (53%) were immunocompromised. All patients were treated in the ICU for an MDR Pseudomonas infection. As shown in table 1, the most common indications were 49% HAP/VAP, 17% complicated intra-abdominal (cIAI), or 17% urinary tract infections (cUTI). 4 (24%) patients had additional treatment courses of CT started empirically when infection was suspected. Median time to initiation for all courses was 2 days after obtaining cultures and median duration was 7 days. 12 patients were on CVVHD (median flow rate 2.5L/hr) and 7 were on iHD. 2 patients received iHD after CVVHD. Median dose while on CVVHD was 1500mg every 8 hours. The median dose on iHD was that approved by FDA for cIAI and cUTI: 750mg x1 followed by 150mg every 8 hours. Clinical success was achieved in 12 (71%) patients and 30-day mortality was 8 (47%).

Table 1: Details on first courses of CT for patients on RRT

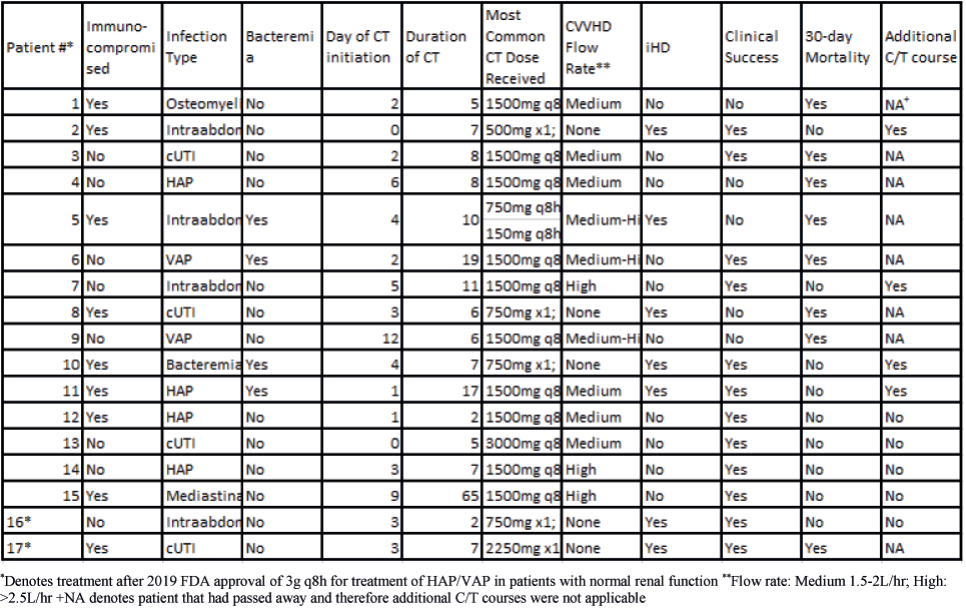

*Denotes treatment after 2019 FDA approval of 3g q8h for treatment of HAP/VAP in patients with normal renal function **Flow rate: Medium 1.5-2L/hr; High: >2.5L/hr +NA denotes patient that had passed away and therefore additional C/T courses were not applicable

**Conclusion:**

This case series provides real-world results of outcomes for critically ill patients on RRT treated with CT. Clinical success rates were similar to other published literature despite the severity of illness of this cohort, which is corroborated by the high 30 day, all-cause mortality. Ultimately, further evaluation of CT dosing in patients on RRT is warranted.

**Disclosures:**

**Laura A. Puzniak, PhD**, **Merck & Co., Inc.** (Employee) **Kelly Harris, PharmD, BCPS**, **Merck & Co. Inc** (Employee) **Trevor C. Van Schooneveld, MD, FACP**, BioFire (Individual(s) Involved: Self): Consultant, Scientific Research Study Investigator; Insmed (Individual(s) Involved: Self): Scientific Research Study Investigator; Merck (Individual(s) Involved: Self): Scientific Research Study Investigator; Rebiotix (Individual(s) Involved: Self): Scientific Research Study Investigator **Scott J. Bergman, PharmD, FCCP, FIDSA, BCPS, BCIDP**, **Merck & Co., Inc** (Grant/Research Support) **Scott J. Bergman, PharmD, FCCP, FIDSA, BCPS, BCIDP**, Merck & Co., Inc (Individual(s) Involved: Self): Research Grant or Support

